# Arthroscopic Bankart Repair With Remplissage Results in Low Recurrent Instability Rates Without Reducing Shoulder Range of Motion at Midterm Follow-up: A Systematic Review of Studies With Minimum 5-Year Outcomes

**DOI:** 10.1177/03635465251324930

**Published:** 2025-09-24

**Authors:** Patrick J. Tansey, David S. Clark, Verdinand C.B. Ruelos, Robert W. Lindeman, Jeremy S. Somerson

**Affiliations:** † Department of Orthopaedic Surgery and Rehabilitation, The University of Texas Medical Branch, Galveston, Texas, USA; ‡ John Sealy School of Medicine, The University of Texas Medical Branch, Galveston, Texas, USA; Investigation performed at The University of Texas Medical Branch, Galveston, Texas, USA

**Keywords:** arthroscopy, Bankart repair, joint instability, remplissage, shoulder

## Abstract

**Background:**

Anterior shoulder instability remains a frequent occurrence in the young, active patient. Arthroscopic remplissage in addition to anteroinferior labral (Bankart) repair is a common treatment to address subcritical bone loss and decrease recurrent dislocation. Despite increasing use, concerns remain about the long-term outcomes of remplissage given its nonanatomic nature and relative novelty.

**Purpose:**

To systematically review published literature to assess functional outcomes, range of motion, recurrence rates, and subsequent revision rates after arthroscopic Bankart repair with remplissage for treatment of anterior shoulder instability with minimum 5-year follow-up.

**Study Design:**

Systematic review; Level of evidence, 4.

**Methods:**

Two independent reviewers performed a literature search based on PRISMA (Preferred Reporting Items for Systematic Reviews and Meta-Analyses) guidelines, using the Scopus, PubMed, and Cochrane Library databases. Clinical studies reporting functional outcomes and recurrence data at a minimum of 5 years after arthroscopic Bankart repair and remplissage for shoulder instability were included for analysis.

**Results:**

Three studies including 144 shoulders met the inclusion criteria. The mean clinical follow-up was 104 months (9 years), with all patients having a minimum 60 months (5 years) of follow-up. Of the included patients, 77% were male and the mean age was 29 years (range, 15-72 years), with 51% participating in contact sports. All patients had glenoid bone loss <25%. The mean Rowe score increased from 49 to 97 (*P* < .001). No difference was found in preoperative versus postoperative forward elevation (171° vs 176°; *P* = .09) or external rotation at 90° of abduction (90° vs 86°; *P* = .09). The overall rate of recurrent instability events was 10%, with 8% of patients having a repeat dislocation, 4% undergoing repeat operation, and 76% returning to sport.

**Conclusion:**

In patients who had recurrent instability with an engaging Hill-Sachs lesion and subcritical glenoid bone loss, arthroscopic Bankart repair with remplissage showed excellent functional outcomes without restricting range of motion in published literature with minimum 5-year follow-up. The recurrent instability and dislocation rates remain lower than the reported rates after isolated Bankart repair. Further reports are needed to determine the clinical outcomes at long-term follow-up.

Anterior shoulder instability is a frequent occurrence in the young, active population.^37^ Anterior shoulder dislocations can be successfully managed nonoperatively or operatively, although recurrent instability remains a common complication, with rates reported as high as 75%.^5,35,44^ The traumatic force of the dislocation event can create anteroinferior glenoid bone loss and a Hill-Sachs lesion of the posterosuperior humeral head, both of which can predispose patients to repeat dislocations and chronic instability if not appropriately addressed.^11,15^ Arthroscopic remplissage, a posterior capsulotenodesis, in combination with anteroinferior labral (Bankart) repair, is a technique described to address patients with subcritical bone loss and associated Hill-Sachs lesions.^46^ Since its introduction in 2004, arthroscopic Bankart repair with remplissage has been shown to provide good short-term results and reduced recurrence while avoiding complications in association with bone block procedures.^8,19,26,31,48^

Despite increasing use of remplissage, concerns remain about its long-term outcomes given its nonanatomic nature and the relative lack of long-term outcome studies.^10^ Although reports remain mixed, several short-term clinical and biomechanical studies have shown the potential for restricted motion, particularly external rotation, after remplissage.^12,16,17,33^ Remplissage, as with all surgical techniques, warrants continued follow-up.

The purpose of this study was to systematically review the literature to assess functional outcomes, range of motion, recurrence rates, and subsequent revision rates after arthroscopic Bankart repair with remplissage for treatment of anterior shoulder instability with minimum 5-year follow-up.

## Methods

**Figure 1.**
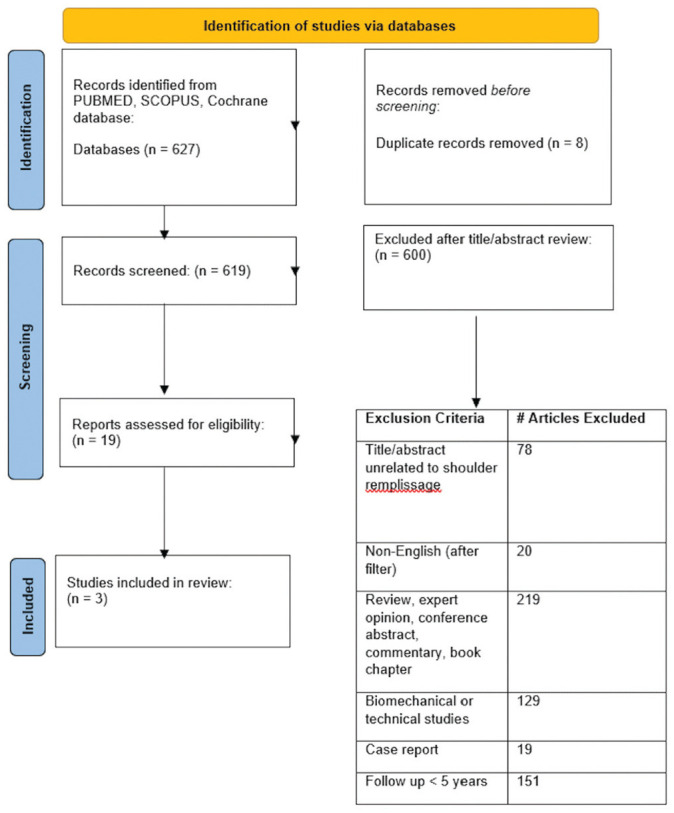
Study selection: PRISMA (Preferred Reporting Items for Systematic Reviews and Meta-Analyses) flowsheet.

A systematic review of the Cochrane Library, PubMed, and Scopus was performed in adherence to the PRISMA (Preferred Reporting Items for Systematic Reviews and Meta-Analyses) guidelines (Figure 1). Two authors completed independent searches. Disagreements regarding study inclusion were determined by the study's senior author (J.S.S.). When overlapping study populations were identified, only the most recent article was included for final review and analysis. Search terms included “Bankart AND remplissage,”“arthroscopic remplissage OR remplissage,”“shoulder instability AND remplissage,” and “remplissage AND long-term.” Level of evidence was determined per the Oxford Centre for Evidence-Based Medicine Guidelines.^36^

Inclusion criteria were clinical studies reporting either clinical or radiographic outcomes after arthroscopic Bankart repair with remplissage with a minimum 5-year follow-up period, studies with level 1 through 4 evidence, studies that included ≥2 patients, and studies published in the English language. Exclusion criteria were studies that failed to report clinical or radiographic outcomes, studies that entailed open procedures, biomechanical or surgical technique papers, scientific meeting or conference abstracts, studies that included <2 patients, and studies published in non-English languages.

Patient demographic data including age, sex, age at first dislocation, number of dislocations, and level of sports participation were collected. Preoperative Instability Severity Index Score (ISIS)^2^ was collected when available. Percentage of glenoid bone loss and size of Hill-Sachs lesion were collected when available. Method of calculations for both glenoid and humeral-sided bone loss was determined independently by the study authors. Operative technique details included patient position and number of anchors used for remplissage. Concomitant procedure data beyond Bankart repair and remplissage were collected if present. Complication types in this review included apprehension, repeat dislocation, need for additional procedure, or other complications reported by the authors. The total complication rate was calculated by summing all reported complications. Repeat instability was defined as a positive postoperative apprehension sign, subluxation, or dislocation event. Repeat dislocation was analyzed separately. Need for repeat stabilization procedure was defined as any continued instability warranting return to the operating room.

Descriptive statistics were weighted based on the sample sizes of each group. Continuous variables were analyzed with a Student *t* test. Frequencies and percentages were reported for categorical data. Categorical variables were analyzed with a chi-square goodness-of-fit test. Statistical analysis was performed using R 4.40 (The R Foundation).

## Results

Three studies met criteria for final analysis, with a total of 144 shoulders that underwent Bankart repair with remplissage (Table 1).^3,6,7^ The mean clinical follow-up was 9 years (range, 5-13 years), with all patients having a minimum of 60 months (5 years) of follow-up. The mean patient age was 29 years (range, 16-50 years). Of these patients, 111 (77%) were male and 33 (23%) were female. Patients experienced a mean of 6 dislocations (range, 1-50) before surgery. Median number of dislocations was unavailable. One study^7^ categorized 22% of patients having <4 dislocations, whereas 78% had ≥4 dislocations. A total of 81 (56%) procedures were performed on the patient's dominant arm. All indicated patients had recurrent instability, had an engaging Hill-Sachs lesion, and lacked substantial glenoid bone loss (<25%; range, 0%-25%). All 3 articles determined engagement of the Hill-Sachs lesion with dynamic arthroscopic assessment intraoperatively,^3,6,7^ with one study^6^ specifying the arm position to be in abduction and external rotation during assessment. Additional inclusion criteria included an ISIS ≥3 in one study^7^ and an ISIS ≥4 in another.^3^ One article did not include method for measuring glenoid or humeral bone loss.^3^ Two studies measured glenoid bone loss with computed tomography using the Sugaya method.^43^ One study measured Hill-Sachs size via axial cuts of magnetic resonance imaging scans but did not specify precise method, listing 34 (52%) as medium-size lesions and 31 (48%) as large.^7^ Another study measured a ratio of the depth of the Hill-Sachs lesion to the radius of the humeral head on computed tomography scan and found a mean of 18% (9%-42%).^6^ All studies confirmed the Hill-Sachs lesion to be engaging by intraoperative dynamic evaluation.^3,6,7^ A total of 106 (74%) patients participated in sports, with 69 (48%) of these participating in contact sports. The mean ISIS was 3 (range, 1-6).

**Table 1:** Study and Subject Characteristics^*a*^

Parameter	Value
No. of studies analyzed	3
Level of evidence	
3	2 (66)
4	1 (33)
Financial conflict of interest	
Yes	00 (0)
No	2 (67)
Not reported	1 (33)
Shoulders, n	144
Male	111 (77)
Female	31 (23)
Mean subject age, y (range)	29.0 (16-50)
No. of dislocations before surgery (range)	6.4 (1-50)
No. of procedures on dominant arm	81 (56)
Glenoid bone loss	
0%-25%	116 (81)
>25%	00 (0)
Not reported	28 (19)
Participation in sports	106 (74)
Participation in contact sports	69 (48)
Mean Instability Severity Index Score (range)	2.7 (1-6)
Mean follow-up, y (range)	8.7 (5-12.5)

a Data expressed as n (%) unless otherwise noted.

Two groups^3,6^ performed the procedure with patients in a beach-chair position, whereas 1 group^7^ used the lateral position. Two groups routinely used 2 anchors for remplissage,^3,6^ whereas the other group^7^ used 1 or 2 anchors on a case-by-case basis. No additional procedures were reported.

The mean Rowe score increased from 49 to 97 (*P* < .001). Two studies reported a final Walch-Duplay score of 87.7 (range, 70-100).^3,6^ Changes in strength were too infrequently recorded to review. At final follow-up, no difference was reported in preoperative versus postoperative forward elevation (171° vs 176°; *P* = .09) or external rotation at 90° of abduction (90° vs 86°; *P* = .09) (Table 2). The mean reachable internal rotation thoracic vertebral level was unchanged from a mean of T10. Of 116 patients, 88 (76%) returned to sport by final follow-up. One study reported data on return to play at the same level (69%).^6^

**Table 2:** Outcomes After Bankart Repair and Remplissage

Parameter	Preoperative	Postoperative	*P*
Rowe score	49	97	<.001
External rotation, deg	90	86	.09
Forward elevation, deg	171	176	.53
Return to sport, %		76	

The overall rate of recurrent instability events was 10% (n = 15), with 8% of cases (n = 11) having a repeat dislocation and 4% (n = 5) undergoing repeat operation (Table 3). Of the 11 dislocations, 9 occurred within the first 13 months and 7 occurred during contact sport activity or trauma. One study^6^ analyzed patients who sustained recurrent postoperative dislocation and found that they were younger (mean 20 vs 23 years; *P* = .04) and had deeper Hill-Sachs lesions (25% vs 18%; *P* = .03). Each of the 5 patients who required an additional stabilization procedure underwent a Latarjet coracoid transfer. Four (3%) other complications included 1 case of transient ulnar nerve palsy, 1 case of mechanical symptoms, 1 case of early fatigue, and 1 case of temperature hypersensitivity. When factoring instability events, repeat operations, and other complications, the overall complication rate was 17% (n = 24).

**Table 3:** Complications and Reoperations

Parameter	n	%
Total complications	24	17
Recurrent instability	15	10
Recurrent dislocation	11	8
Revision stabilization procedure	5	4
Transient ulnar nerve palsy	1	1
Mechanical symptoms	1	1
Early fatigue	1	1
Hypersensitivity	1	1

Due to limited literature availability and stringent inclusion criteria, only a small number of studies were available for analysis after systematic review. However, our literature search identified 6 additional studies that had a mean, but not minimum, follow-up of >5 years.^4,13,28,30,32,40^ The results of 5-year mean and minimum follow-up studies were pooled for exploratory post hoc descriptive analysis. An additional 307 patients were included in this analysis for a total of 502 patients. Mean age was 28 years (range, 15-72 years). The mean follow-up was 7 years (range, 2-15 years). Preoperative forward elevation and external rotation at 90° of abduction were 168° and 82°, respectively. Postoperative forward elevation and external rotation at 90° of abduction were 173° and 82°, respectively. The Rowe score improved from 49 to 93. The repeat instability rate was 6% (n = 32), and the dislocation rate was 5% (n = 23). Return to sport was possible for 88% (291 of 332) of patients. Twelve additional reoperations were detected in pooled analysis, with a reoperation rate of 3% (n = 17). Two patients underwent arthroscopic debridement for stiffness, 8 patients underwent a Latarjet procedure, 1 patient underwent revision Bankart repair with remplissage, and 1 patient was converted to a total shoulder arthroplasty.

## Discussion

The purpose of this systematic review was to evaluate the outcomes and complications of arthroscopic Bankart repair with remplissage at a minimum 5-year follow-up. Our goal was to provide clinical insights and facilitate evidence-based discussions on the efficacy and safety of a relatively new nonanatomic procedure. The most important finding was that at midterm follow-up, Bankart repair with remplissage had a 90% successful stabilization rate without significant loss of shoulder range of motion in patients with recurrent instability, an engaging Hill-Sachs lesion, and subcritical glenoid bone loss.

Indications for remplissage remain unclear.^8^ All patients from this review had a history of recurrent instability, glenoid bone loss <25%, and an engaging Hill-Sachs lesion. Recent studies have reported low rates of recurrent dislocations and high return to sport after combined Bankart remplissage in patients with engaging Hill-Sachs lesions, nonengaging Hill-Sachs lesions, and first-time dislocations with concern for future instability.^39,40,45^ Pawłuś et al^39^ found a recurrent instability rate of 5% in a review of 7 studies reporting 2-year outcomes after combined Bankart remplissage compared with 30% in Bankart repair in patients with an engaging Hill-Sachs lesion and <25% glenoid bone loss. Despite heterogeneity in bone loss measurement methods between studies in the current analysis and Pawłuś’s report, their 5% recurrent instability rate appears comparable with the 10% recurrence seen at 5 years in the current review. Due to the relatively narrow indications in our included studies, we are unable to draw specific conclusions on the ideal indications and contraindications for combined Bankart remplissage. Although paradigms around shoulder instability continue to evolve, consistency in quantitative bone loss method and categorization of subcritical bone loss by future investigators will be critical to better understand which injury patterns benefit most from additional remplissage long-term.^42,48^

The clinical outcomes of Bankart repair at mid- and long-term follow-up have previously been reported. Harris et al^21^ reported the outcomes of arthroscopic or open Bankart repair at minimum 5-year follow-up. Final Rowe score was 87, and 74% of patients returned to sport at the same level of play. Murphy et al^34^ later reported Bankart repair outcomes at minimum 10-year follow-up, finding a final Rowe score of 87, with a 77% rate of return to play. Similarly, Hurley et al^25^ reported a final Rowe score of 89, with 76% of patients returning to same-level play 10 years after Latarjet. The final Rowe score after Bankart remplissage in our review was 97, which appears favorable in comparison to that of isolated Bankart repair or bone block transfer. The superior outcome scores seen in this review may be attributed to reduced positional apprehension and instability rates after remplissage, as these have been shown to be a predictor of successful 5-year outcomes.^24^ However, it is unclear whether improved Rowe scores translate to improved athletic participation, because return-to-sport levels appear consistent across all stabilization procedures.^21,25,34^ Radiographic evidence of instability arthropathy after stabilization procedures is a potential long-term adverse outcome that has previously been reported in 60% and 36% of cases 10 years after Bankart repair and Latarjet, respectively.^25,34^ Radiographic outcomes were not described in our included studies, although this may be an area for future investigators to report.

Despite increasing use of combined Bankart repair and remplissage, concerns about loss of motion after the procedure remain. Historically, nonanatomic stabilization procedures such as the Putti-Platt, Magnusson-Stack, and anterior capsulorrhaphy fell out of favor due to iatrogenic reduction of motion, increased joint stiffness, and subsequent development of glenohumeral osteoarthritis.^29,30^ Elkinson et al^12^ found that the addition of remplissage decreased shoulder rotation by 15° in adduction and increased joint stiffness compared with Bankart repair alone in cadaveric studies. In contrast, Argintar et al^1^ performed a cadaveric study and did not report reduction in motion after addition of remplissage but found that the apex of the humeral head shifted posteriorly and inferiorly at 60° of abduction and maximum external rotation. Differences in biomechanical outcomes may be explained by differences in surgical technique, as Feng et al^14^ found that medially placed anchors and use of multiple anchors decreased humeral head displacement in a finite element motion analysis.

Some clinical studies have also found early loss of shoulder rotation motion after remplissage,^16,22^ although other authors found no difference.^23,45^ MacDonald et al^31^ reported the 2-year results of a level 1 clinical trial in which patients with recurrent instability and an engaging Hill-Sachs lesion were randomized to Bankart repair alone or Bankart repair with remplissage. At 1 year, patients in the remplissage group had significantly decreased external rotation in abduction (75° vs 86°; *P* < .003), although motion in the remplissage group was equal to that of isolated Bankart repair at 2 years. The 4-year results of this randomized trial were recently published, although range of motion at midterm follow-up was not reported.^47^ At minimum 5-year follow-up, we found that mean forward elevation was 176° and mean external rotation was 86°, neither of which was significantly different from preoperative values. These values are comparable with those reported in isolated Bankart repair series with long-term follow-up.^9,27^ Similarly, data from the post hoc exploratory analysis of articles^4,13,28,30,32,40^ with mean follow-up of 5 years suggest that range of motion is not diminished compared with the patient's preoperative state, although it should be noted that 2 patients in one study^32^ required arthroscopic debridement for stiffness. Continued publication of midterm and long-term follow-up may reveal whether changes in glenohumeral joint stiffness and kinematics lead to changes in motion over time.

Recurrent instability after anterior stabilization procedures remains a concern. We found that the overall rate of recurrent instability at 5-year minimum follow-up was 10%, with 8% having a repeat dislocation and 4% undergoing repeat operation. Woodmass et al^47^ reported the 4-year outcomes of a previously published randomized controlled trial comparing isolated Bankart repair versus Bankart and remplissage. The rates of instability and dislocation after remplissage in their study are identical to the rates reported in the current systematic review. Notably, the instability and dislocation rates of isolated Bankart in the report by Woodmass et al were 30% and 22%, respectively. Patients were excluded from their trial if glenoid bone loss exceeded 15%, whereas several included patients in the current analysis had glenoid bone loss 16% to 20%. Addition of remplissage appears to result in a high success rate at midterm follow-up across a wide range of glenoid bone loss, as has previously been reported at short-term follow-up.^22^ Importantly, the risk of recurrent instability appears to remain comparable, if not superior, to isolated Bankart repair. The rate of instability after isolated Bankart repair in studies with >5-year follow-up ranges from 11% to 32%.^21,24,34,38^ Remplissage appears to be particularly beneficial to patients with engaging or “off-track” Hill-Sachs lesions. All patients in the current review were found to have an engaging Hill-Sachs on dynamic assessment. Schwihla et al^41^ found that patients with these findings treated with isolated Bankart repair had a 74% recurrence rate and 48% reoperation rate. Some surgeons may prefer to perform a Latarjet in a high-risk patient. Although comparative studies beyond short-term follow-up are lacking, Bankart repair with remplissage appears to have a success rate comparable with Latarjet at mid- to long-term follow-up.^18,25^ Although the time and the implants required to perform remplissage inherently add cost to the procedure, it is unclear whether the reduced rate of recurrent instability would lead to net cost savings for health systems. Future investigators may consider performing a cost analysis of Bankart repair and remplissage versus alternative treatments to better understand this relationship.

One proposed benefit of Bankart repair with remplissage over bone block procedures for instability cases in patients with subcritical bone loss is a lower rate of complications.^20,22,48^ Haroun et al^20^ performed a systematic review of studies comparing Bankart repair with remplissage versus Latarjet for patients with subcritical bone loss and an engaging Hill-Sachs lesion. Those investigators reported similar functional outcomes and recurrent stability between the 2 procedures but noted that Latarjet had a 7-fold greater complication rate. Gouveia et al^19^ found similar results in a systematic review that also included patients undergoing bone block transfer with either iliac crest or distal tibia allograft. Overall, the reported complication rate after bone block procedures ranges from 6% to 66%.^19,20,22,48^ In our review, 4 patients (3%) had complications unrelated to recurrent instability. This is slightly more than the 1% described in the 2-year outcomes report of a randomized clinical trial.^44^ This may reflect differences in reporting protocols or increased discovery of complications with prolonged follow-up. Bankart repair with remplissage appears to be an effective procedure associated with a lower complication rate than bone-block procedures at 5-year follow-up.

The current systematic review had some limitations. Due to stringent inclusion criteria and few published studies, only 3 retrospective studies met criteria for final analysis, all of which were retrospective level 3 and 4 evidence. Therefore, our results are inherently limited by the biases to which retrospective studies are subject. Although the small number of studies found during systematic review of the literature precludes a more robust analysis, we believe that it also highlights an important shortcoming in our understanding of midterm outcomes of an increasingly performed and researched procedure. The lack of a comparison group to alternative stabilization procedures such as isolated Bankart repair is an inherent limitation of these data. Continued communication of results from a previously reported level 1 randomized trial comparing Bankart repair with versus without remplissage will certainly provide valuable clinical insights.^31,47^ We would encourage the authors to assess range of motion presented in the 2-year outcomes report but absent in the recent 4-year outcomes study. Furthermore, 6 additional studies^4,13,28,30,32,40^ were identified with a mean, but not minimum, 5-year follow-up. The orthopaedic community would benefit from continued follow-up of these cohorts, as doing so could triple the current body of literature regarding minimum 5-year follow-up. An additional limitation of this review is the lack of comparison between Bankart remplissage and alternative stabilization procedures. As the volume of literature increases, future investigators may be able to perform comparative analyses of outcomes after isolated Bankart repair with Bankart remplissage. Lack of consistent reporting of factors such as baseline activity level, quantitative bipolar bone loss, presence of associated injuries, anchor position, and repair technique potentially confounded outcomes. We were unable to determine the median number of dislocations before surgical intervention or perform outcome analysis stratified by number of preoperative dislocations. The small sample sizes of the included studies likely introduced variability due to differences in patient factors, rehabilitation protocols, and selected outcomes—all of which potentially weaken the external validity of our findings. Increased use of validated outcome scores would be beneficial in future works. Finally, the studies included in our systematic review were authored from academic centers by early adopters of remplissage, and their results may not be reproducible in all practice models. Despite these limitations, this systematic review provides relevant insights on an increasingly performed procedure for a common condition and highlights the need for more literature reporting long-term outcomes.

## Conclusion

In patients with an engaging Hill-Sachs lesion and subcritical glenoid bone loss who experienced recurrent instability, arthroscopic Bankart repair with remplissage showed excellent functional outcomes without restricting range of motion in published literature with minimum 5-year follow-up. The recurrent instability and dislocation rate remains lower than the reported rates after isolated Bankart repair. Further reports are needed to determine the clinical outcomes at long-term follow-up.
